# Retraction Note: Urachal tumour: case report of a poorly understood carcinoma

**DOI:** 10.1186/s12957-021-02294-3

**Published:** 2021-06-29

**Authors:** Stefano Scabini, Edoardo Rimini, Emanuele Romairone, Renato Scordamaglia, Luigi Vallarino, Veronica Giasotto, Carlo Ferro, Valter Ferrando

**Affiliations:** 1 Department of Emato-Oncology, AOU San Martino Hospital, Genoa, Italy; 2 ASL 3, Genoa, Italy; 3 Department of Radiology, AOU San Martino Hospital, Genoa, Italy

**Retraction Note: World J Surg Onc 7, 82 (2009)**

**https://doi.org/10.1186/1477-7819-7-82**

The Editor-in-Chief has retracted this article because of overlap with an article by Fujiyama et al [1].

The authors have not responded to any correspondence regarding this retraction.
